# Training Thoracic Surgery Residents in Robotic Surgery: Our Experience

**DOI:** 10.1016/j.atssr.2022.09.006

**Published:** 2022-09-21

**Authors:** Yaacov Abramov, Sharbel Azzam, Calanit Korin, Nachum Nesher, Marina Kolodii, Ashraf Abu Hadwan, Aamer Abu Ammar, Michael Peer

**Affiliations:** 1 Department of Thoracic Surgery, Tel Aviv Medical Center, Tel Aviv, Israel, affiliated with the Sackler School of Medicine, Tel-Aviv University, Tel Aviv, Israel; 2 Sagol Medical Center for Hyperbaric Medicine, Shamir Medical Center, Beer Yaakov, Israel, affiliated with the Sackler School of Medicine, Tel-Aviv University, Tel Aviv, Israel

The da Vinci robotic console (Intuitive Surgical) was approved for thoracic surgery in 2001, with 1.5 million robotic surgical procedures performed up to 2015.^1^ The advantages of robotic over the conventional video-assisted minimally invasive technique are considerable because of the complexity of certain anatomic compartments, such as the mediastinum, but it requires an experienced robotic thoracic surgeon and customized operating room facilities. Robotic training programs focus on reducing the learning curve for acquiring the technical skills required of thoracic robotic surgeons.^2^ The earlier the residents in thoracic surgery are exposed to robotic training, the sooner they may achieve competency as an assistant and later as a console robotic surgeon.^3^ Robotic surgery in not a part of a standard residency program in thoracic surgery in Israel. Our team created a unique curriculum for teaching robotic surgery based on the principles of open mediastinal surgery. A robotic thymectomy was the ideal choice to serve as the inaugural procedure because it requires basic surgical skills, such as dissection, retraction, hemostasis, and others. The end point of our program was the resident's ability to operate as a console surgeon. We present here the results of our robotic surgery program for thoracic surgery residents.

## Preparation

The first robotic thoracic surgery program in Israel was initiated at the Shamir (formerly Assaf Harofeh) Medical Center in 2013 and transferred to the Tel Aviv Medical Center in 2019. The first step was the training of a mentor surgeon (M.P.), who completed the following courses: a robotic thoracic surgery training course in Los Angeles, California, in June 2012 conducted by Prof Kemp Kernstine; a robotic thymectomy course in Berlin, Germany, in March 2013 coordinated by Prof Jens C. Ruckeurt; and a da Vinci System Training Course at the da Vinci Training Center in Paris, France, in August 2013. The report of the first Israeli experience with robotic mediastinal surgery was published in the *Israel Medical Association Journal* in 2018.^4^ The development of an experienced thoracic robotic surgeon (M.P.) enabled us to proceed to creating the teaching program in thoracic robotic surgery. It continued by the training of an assistant surgeon (S.A.) and divided into an “assistant” step and a “console” step. In the assistant step, the resident was required to complete an overview of the robotic system to become familiarized with the fundamentals of the robotic platform and to acquire the basic technical skills required for its operation. This included training in virtual reality and simulation, consisting of EndoWrist (Intuitive Surgical) manipulation, camera and clutching, needle control, suturing, energy application, and dissection. A passing score of 80% for each module was necessary for qualification as a bedside assistant surgeon. The responsibility of the resident (S.A.) at that level included docking the robotic trocars and safe introduction of robotic instruments; communication with the console surgeon (M.P.); and assistance during the actual surgical procedure, including suctioning, retraction, and hemostasis. A learning curve of 15 to 20 robotic procedures was required to complete the assistant step and to progress to a console surgeon. The surgical procedures at this step included anterior mediastinal robotic surgery consisting of thymectomy for myasthenia gravis, thymectomy for thymoma without myasthenia gravis or other anterior mediastinal tumor resection, and aortopulmonary window tumor or lymph node biopsy (Figure).^1^, ^2^, ^3^, ^4^ The candidate resident’s competence was measured by guidelines set by the Global Evaluative Assessment of Robotic Skills^5^ or by the Objective Structured Assessments of Technical Skill.^6^ By developing a second console surgeon (S.A.), the program proceeded with training of new assistant surgeons, according to the same principles.

**Figure:**
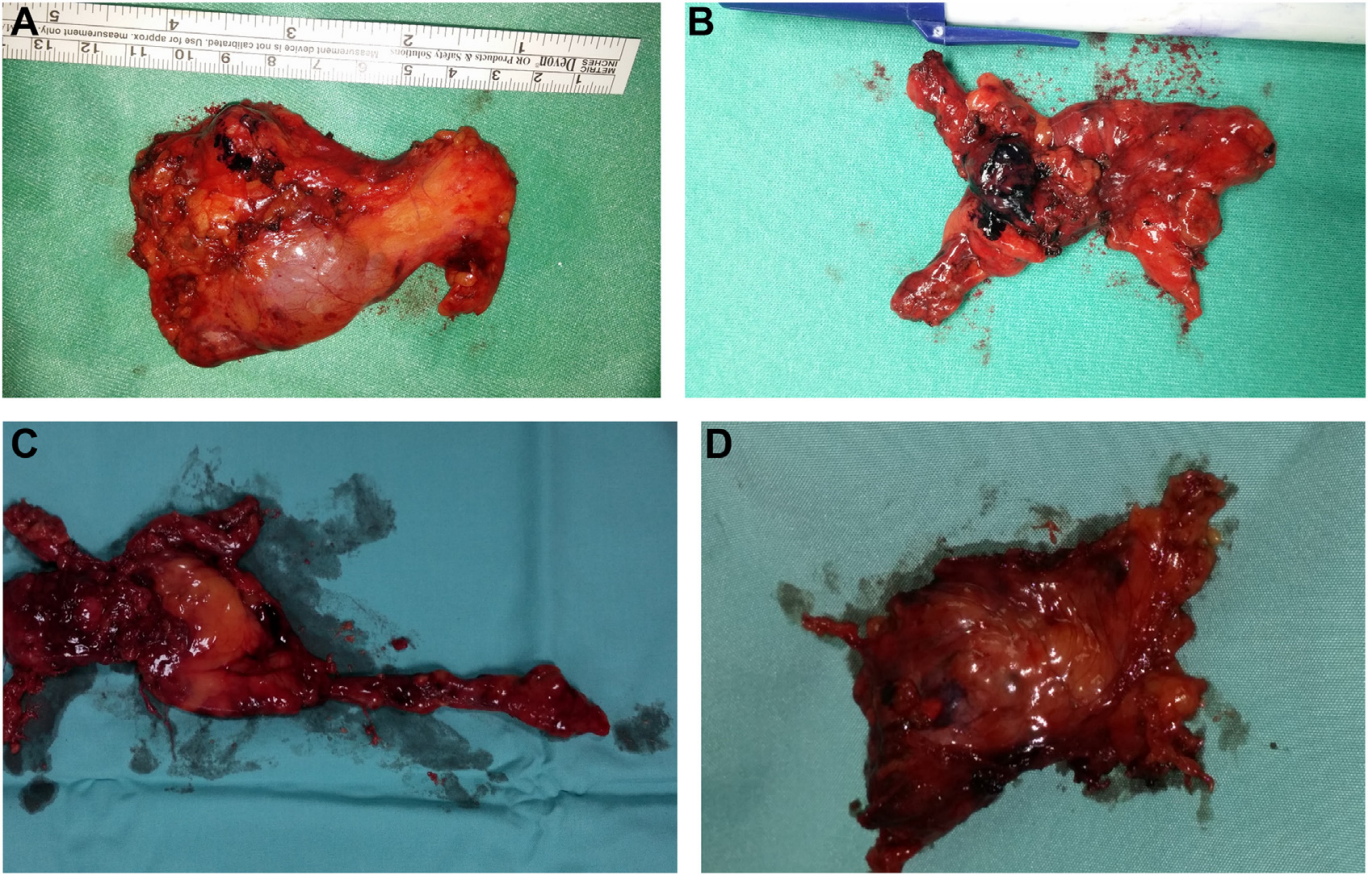
Robotically removed thymoma. (A) First example. (B) Second example. (C) Third example. (D) Fourth example.

In cases of anterior mediastinal tumors, preoperative computed tomography demonstrated features highly suggestive of thymoma—generally smaller than 6 cm in diameter and free of signs of invasion of the surrounding structures. The criteria for selection of patients thought to have thymoma followed the recommendations of Cheng and coworkers^7^ and included an encapsulated tumor in the anterior mediastinum, distinct fat plane between the tumor and mediastinal organs without signs of compression, and unilateral tumor predominance in tumors >3 cm. In patients with myasthenia gravis without thymoma, the entire mediastinal thymic tissue was dissected robotically. The selection of cases appropriate for teaching of residents was based on the following parameters: size of the thymoma, proximity of the thymoma to the phrenic nerve, and relationship of the thymoma to the innominate vein. Specifically, tumors <3 cm that were distant from phrenic nerves or the innominate vein were operated on by training residents.

## How I Teach it

We began our program by planning of the operation by the mentor surgeon, console surgeon, and assistant surgeon, taking into account the following considerations:

1. The preoperative choice of the side of surgical procedure and port placement is based on computed tomography findings:
  a. Left-sided operation facilitates dissection of larger thymic tissue below the left innominate vein and in the aortopulmonary window.
  b. Right-sided operation provides an easier learning curve because of a larger operative field accomplished by heart positioning.
2. Patient positioning consists of either left or right side up to a 15° to 30° angle for better visualization of the contralateral phrenic nerve, with the arm partially extended to avoid damage to the brachial plexus. The chest is prepared for possible sternotomy in all cases.
3. A 3-arm approach by the 3-arm da Vinci Surgical System is used for all anterior mediastinal surgeries. The technique includes dissection of the 4 thymic poles that border the pericardium caudally, the innominate vein cranially, and the phrenic nerves laterally.
  a. The responsibilities are divided as follows:
    i. The program director together with the assistant surgeon is responsible for patient positioning, incisions, camera and port placement, docking, and assistant surgeon for instrument insertion and manipulation (exchanging, suctioning, hemostasis, and retraction).
    ii. One of the key aspects of training is the safe introduction of ports and robotic instruments into the chest and manual collaboration during surgery. The first 15-mm camera port is inserted at the fifth intercostal space in the anterior axillary line (a pectoralis muscle is retracted medially, and the incision is positioned lateral to the breast). A 3-dimensional 30° video endoscope is introduced through the camera port, followed by carbon dioxide insufflation (in steps up to a pressure of 8 to 10 mm Hg).
    iii. Two other 8-mm ports are placed at the third intercostal space and at the eighth intercostal space in the midaxillary and midclavicular lines. An additional 5-mm accessory port, generally for the purpose of suctioning, is made at the seventh intercostal space in the anterior axillary line in selected cases.
    iv. The console surgeon’s responsibilities include the identification, dissection, and grasping of thymic horns, thymic fat, or mediastinal tumor by means of the robotic Maryland bipolar forceps (right hand) and the Cadière forceps (left hand; EndoWrist).
    v. The mentor surgeon is responsible for guiding the console surgeon through the following steps:
      [1] dissection of the ipsilateral inferior thymic horn, allowing a distance of 5 mm from the phrenic nerve;
      [2] dissection of the contralateral thymic horn without entering the contralateral pleural space;
      [3] retraction of the thymic and anterior mediastinal fat (with or without the thymoma or any other mediastinal tumor), dissection from the pericardium and mediastinal great vessels and mobilization of 2 superior cervical horns on the innominate vein, and ligation or clipping of the thymic veins; and
      [4] en bloc mobilization and removal of the tumor with the Endo Catch 10-mm pouch (Universal Intermodal Services) by the assistant surgeon through the widened lower port incision. A 28F or 32F chest tube is inserted through the accessory port or the lower port incision. Sutures are closed.

In total, 120 patients (58 men [48.3%] and 62 women [51.7%], with a mean age of 56.5 years [range, 18-88 years]) underwent robotic mediastinal surgical procedures in 2 Israeli medical centers from October 2013 to October 2021. Sixty-four patients (53.3%) were operated on at the Shamir (formerly Assaf Harofeh) Medical Center and 56 patients (46.7%) at the Tel Aviv Medical Center. The robotic surgical procedure was left sided in 81 patients (67.5%) and right sided in 39 patients (32.5%). Anterior mediastinal robotic surgery as a part of our program was performed in 102 patients (85%) with no perioperative deaths and 3.3% conversion rate (4 patients). Five patients (4.2%) underwent intrapericardial resections. Average blood loss was 15.5 mL (range, 0-300 mL). Postoperative complications occurred in 13 patients (10.8%), which included pneumonia in 3 patients (2.5%); moderate pleural effusion that required drainage in 3 patients (2.5%); phrenic nerve palsy in 3 patients (2.5%); and pulmonary emboli, empyema, esophageal anastomotic leak, and delirium in 1 patient each (0.8%). The mean operative time was 103 minutes (range, 45-380 minutes), and the mean hospital stay was 4.2 days.

Of the 64 robotic surgical procedures at the Shamir Medical Center, 25 were performed by a training resident (today a practicing robotic thoracic surgeon, S.A.) and 39 by the mentor surgeon (program director, M.P.). Only 15 of the most complicated cases among the 56 robotic surgeries at the Tel Aviv Medical Center were performed by the program director; the others (41) were performed by trainees.

## Comment

The field of robotic thoracic surgery continues to grow both in volume and in the complexity of surgeries, establishing the need to develop resident robotic surgery programs in teaching hospitals to contend with the greater numbers of patients. Here, we analyzed our experience of training thoracic surgery residents in our robotic mediastinal surgery training program. The robot, with arms that can be articulated with 7 degrees of freedom and 360 degrees of rotation, is perfectly suited for operations in narrow spaces, such as the mediastinum, where there is great risk of damage to major blood vessels or nerves. Paradoxically, surgical procedures of the mediastinum are the most frequent procedures carried out in the initial phase of the learning curve because that anatomy represents an ideal training model for executing the basic procedures of thoracic surgery.

Our initial goals of resident training for bedside assistant and console surgeon are minimizing risks and ensuring patient safety. The da Vinci robotic surgical system enables surgeons to perform accurate and safe dissection of the left thymic lobe from a right-sided approach and of the right thymic lobe from a left-sided approach by EndoWrists with articulated movements that permit improved maneuverability in the operating field. The EndoWrist robotic technique simulates normal wrist movements and improves safety and precision in performing complex procedures.

In our series, there were no deaths, the conversion rate was low, and the postoperative hospital stay together with postoperative complications was acceptable. As experience grew, a shift in the patient population was seen toward more complex thoracic surgical procedures being performed only by experienced training surgeons. Two residents completed our robotic residency program in mediastinal thoracic surgery, and 2 new residents recently enrolled into the program.

One important advantage of our robot-assisted thoracic surgery program is its quick adaptation to the operating surgeon’s techniques. A survey of 16 program directors in robotic surgery found that they believed the trainees must perform at least 10 procedures as a primary bedside assistant during residency.^3^ We are aware that the main limitations of introducing a robotic thoracic surgery training program in hospitals are the few available mentor robotic surgeons and the need for specialized operating room facilities. Our program protocol recommends that thoracic surgical residents operate on 10 to 15 patients with the mentor surgeon as assistant surgeon for them to obtain full privileges, with a minimum of 10 cases to attain console surgeon status.

Our study includes 120 patients operated on during a period of 8 years in the only department in the country permitting opportunity for residents in robotic thoracic surgery training. The study results demonstrated that 2 residents completed the robotic residency program and 2 new residents enrolled in the program. Our program for training thoracic surgical residents to skillfully apply a robotic system is safe and effective for the surgical treatment of mediastinal pathologic processes. We are able to achieve competency in mediastinal robotic surgery and to educate a new generation of competent thoracic robotic surgeons.
